# The influence of climate and population density on *Buxus hyrcana* potential distribution and habitat connectivity

**DOI:** 10.1007/s10265-023-01457-5

**Published:** 2023-04-28

**Authors:** Shirin Alipour, Łukasz Walas

**Affiliations:** Institute of Dendrology, Polish Academy of Sciences, Parkowa 5, 62-035 Kórnik, Poland

**Keywords:** Global warming, Habitat fragmentation, Hyrcanian forests, MAXENT, Species conservation

## Abstract

Changes in environmental factors, human impact, and interactions between them accelerate the extinction of woody species. Therefore, conservation programs are needed to protect endangered taxa. However, the relationship between climate, habitat fragmentation, and anthropogenic activities and their consequences are still not well understood. In this work, we aimed to evaluate the impact of climate change and human population density on the *Buxus hyrcana* Pojark distribution range, as well as the phenomenon of habitat fragmentation. Based on species occurrence data throughout the Hyrcanian Forests (north of Iran), the MAXENT model was employed to estimate the potential distribution and suitability changes. Morphological-spatial analysis (MSPA) and CIRCUITSCAPE were used to assess habitat fragmentation and its connectivity. According to the main results obtained from future scenarios, the potential range will significantly decrease due to the lack of suitable climatic conditions. Meanwhile, *B. hyrcana* may not be able to shift in potentially suitable areas because of human influence and geographic barriers. Under RCP scenarios the extent of the core area would be reduced and the edge/core ratio significantly increased. Altogether, we found negative effects of the environmental change and the human population density on the continuity of habitats of *B. hyrcana*. The results of the presented work may improve our knowledge connected with in situ and ex situ protection strategies.

**Supplementary Information:**

The online version contains supplementary material available at 10.1007/s10265-023-01457-5.

## Introduction

Dense forests of the Hyrcanian region in North Iran constitute an important refugium for biodiversity and are very rich in endemic plant species (Ghorbanalizadeh and Akhani 2022; UNESCO World Heritage Centre 2019). This green belt between the Caspian Sea and the Alborz Mountains is a part of the Euro-Siberian phytogeographic region and contains many Arcto-Tertiary relict elements, which are remnants of the ancient flora that once covered vast areas of Europe (Akhani et al. 2010; Sagheb-Talebi et al. 2014).

**Fig. 1:**
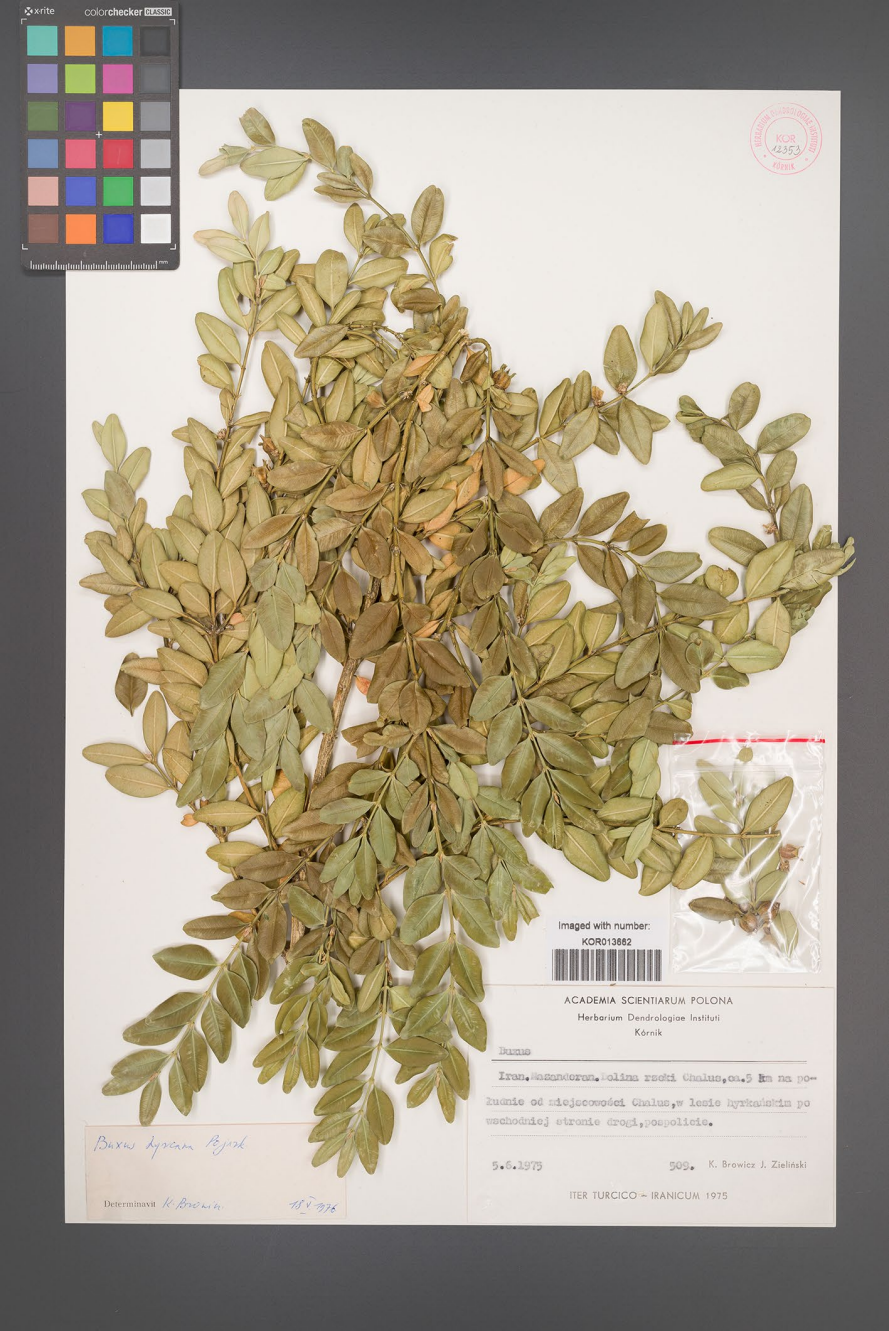
Herbarium sheet (KOR 12353) from the Herbarium of the Institute of Dendrology, Polish Academy of Sciences with a specimen of *Buxus hyrcana*, collected by K. Browicz and J. Zieliński. Available digitally at: https://rcin.org.pl/dlibra/publication/204311

One of the important tree species and survivors from the Pliocene epoch in the Hyrcanian forests is the Caspian boxwood tree *Buxus hyrcana* Pojark (Fig. 1). It is an evergreen, slow-growing, long-lived, and shade-tolerant species, which can grow as a shrub or a tree up to 12 m tall. *Buxus* occupies mainly the lowland forests (20–400 m a.s.l.), rarely exceeding 1000 m a.s.l. (Sagheb-Talebi et al. 2014). It is usually found as part of the understory in the Querco-Buxetum community, where it co-occurs with such species as *Quercus castaneifolia* C.A.Mey., *Alnus glutinosa* L., *Acer* spp., *Pterocarya fraxinifolia* (Lamb.) Spach, *Diospyros lotus* L., *Albizia julibrissin* Durazz. and *Gleditsia caspica* Desf. (Mobayen 1970). The taxonomic position of this taxa is not entirely clear and it is often treated as a subspecies, *Buxus sempervirens* subsp. *hyrcana* (Pojark.) Takht. Between *B. hyrcana* and *B. sempervirens* sensu stricto occurs *Buxus colchica* Pojark., treated as another part of the *B. sempervirens* complex. Such a pattern with closely related Euxinian and Hyrcanian taxa is observed also for genera *Ilex* L. and *Hedera* L. (Ghorbanalizadeh and Akhani 2022).

Boxwood is listed in the “endangered” category in the Red Book of Iran (Jalili and Jamzad 1999). Long-term local anthropogenic activity is largely responsible for the deforestation and fragmentation of the lowland Hyrcanian forests (Akhani et al. 2010; Leroy et al. 2011; Mahjoori 2002; Ramezani et al. 2008). The habitats of valuable trees that have been located in the lowlands are faced with overharvesting problems. Habitats of *B. hyrcana* are no exception to this principle since the *Buxus* was very often logged because of its valuable timber (Jalili and Jamzad 1999). Besides, boxwood blight disease and box-tree moth, which became a growing problem, are additional causes of boxwood stands degradation (Salehi Shanjani et al. 2018). These three main factors, and in parallel urbanization, afforestation, and increased demand in the timber market, have brought not only *B. hyrcana* habitat, but also Hyrcanian lowland forests as a whole to the brink of total destruction.

Understanding the links between plant species distribution and environmental factors provides new information on ecosystem stability, allowing us to estimate where species will occur in the future (Landi et al. 2018; Wisz et al. 2013). The climate is an influential factor in terms of regulating spatial patterns of species distribution (Ge et al. 2021; Wisz et al. 2013). Ranges of individual plant taxa make up the entire vegetation, which is a crucial component of the Earth's terrestrial biosphere (Franklin et al. 2016). Today, climate change and human activities are accounted as main factors leading to vegetation changes (Ge et al. 2021; Yang et al. 2022). Quantifying the impact of these factors is now a key challenge that must be addressed in the face of ongoing land degradation and habitat fragmentation. This can allow development of strategies to cope with environmental crises (Jiang et al. 2017, 2020; Zheng et al. 2019). In recent years, many researchers have studied this field on the global, continental, national, and regional scales (Cannone et al. 2021; Cao et al. 2021; Gang et al. 2017; Ge et al. 2021; Li et al. 2020).

Hyrcanian forests have been identified as a sensitive and endangered area due to climate change, which is connected with the possibility of a temperature increase of about 4.3 °C by 2070 (Alavi et al. 2019; Alipour et al. 2022; Mahmoodi et al. 2023; Taleshi et al. 2019). Recent studies have found the negative impact of climatic instability on the ranges of many tree species in this region, for example, *Ulmus glabra* Huds. (Mohammadi et al. 2019), *Taxus baccata* L (Alavi et al. 2019; Mahmoodi et al. 2023), *Quercus castaneifolia* C.A.M (Taleshi et al. 2020), *Populus caspica* Bornm (Alipour et al. 2022) and *Gleditsia caspica* (Yousefzadeh et al. 2022). Parallel with climate change, strong human pressure could pose a major threat to the unique biological heritage of northern Iran (Lahijani et al. 2008; Leroy et al. 2011; Lowe et al. 2005; Shanjani et al. 2010; Young et al. 1996). The Iranian Parliament adopted the Five-Year Development Plan (FYDPs) and further, Forest’s Breathing Plan (FBP), aiming at conserving natural resources and the environment (Goushehgir et al. 2022; Mohadjer and Feghhi 2020). However, it is still possible to observe a decrease in the area of Hyrcanian forests and an increase in the exploitation of forest resources (Sagheb-Talebi et al. 2014). Therefore, forward-looking management requires predictive systems (Goushehgir et al. 2022) focusing on potential range shifts of endangered species from this region to provide information for efficient planning for their protection in the future (Ahmadi et al. 2020; Alipour et al. 2022; Song et al. 2021; Taleshi et al. 2019).

Designing an effective conservation strategy for an endangered species, whether ex situ or in situ, is dependent on a thorough understanding of its environmental requirements. The SDM methods (Species Distribution Modelling), such as MAXENT software, use environmental data together with localization of species to determine areas suitable for species (Phillips et al. 2006). Thus, it is possible to understand the evolution of a species' range, identify potential refugia, or quantify the impact of future climate change on existing populations (Sękiewicz et al. 2020, 2022; Tekin et al. 2022; Varol et al. 2022a; Walas et al. 2019). In parallel to modeling potential range, assessing connectivity between known stands allows for the identification of barriers and the evaluation of the impact of anthropogenic landscape modifications (de Oliveira-Junior et al. 2020; Nor et al. 2017; Zetterberg et al. 2010), which should be considered a critical factor affecting a variety of ecological phenomena (Fagan and Calabrese 2006; Schloss et al. 2012). Maintaining and restoring landscape connectivity is essential to counter fragmentation, and is one of the conservation planning tools for prioritizing areas (Beier et al. 2006; Choe et al. 2021; Correa Ayram et al. 2017). Therefore, it is important to consider this variable in the context of climate change adaptation (Brost and Beier 2012).

Populations of the same species located in different, isolated regions may differ in their response to climatic factors, as has also been repeatedly observed for woody species (Song et al. 2021; Taib et al. 2020). *B. sempervirens* sensu lato may be considered an example of a disjunct range taxon, and populations classified as *B. hyrcana* may be considered a group of populations with distinct environmental requirements. Several works that focus on determining the potential range of woody species from Iran have already been published (Ahmadi et al. 2020; Alavi et al. 2019; Alipour et al. 2022; Haidarian Aghakhani et al. 2017; Taleshi et al. 2019). There is also an article on the potential range of *B. sempervirens* in Turkey (Varol et al. 2022b). Although there exist some works on the distribution of boxwood in Iran (Esmailzadeh and Soleymanipor 2016; Soleymanipour and Esmailzadeh 2015), so far analysis of the potential range of this species has not been conducted. In this study, a potential range of *B. hyrcana* is determined by its bioclimatic niche, but also takes into account the linkage between the degree of connectivity and the landscape functions. Thus, based on occurrence data the objectives of this study were defined as follows: (1) modeling the ecological niche to detect the potential species distribution for the entire Hyrcanian forests; (2) and at the same time considering morphological spatial pattern analysis (MSPA) and structural connectivity to illustrate potentially patches of suitable habitat and structural corridors connected.

## Materials and methods

**Fig. 2:**
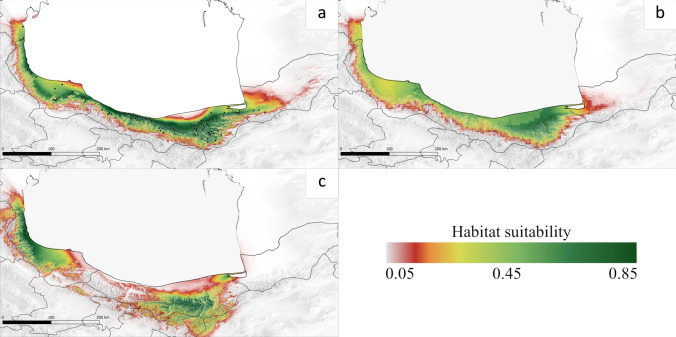
Predicted potential range of *Buxus hyrcana* under current and past climatic conditions. **a** Current range with 80 species occurrence records in North of Iran (black dots), **b** Late Holocene, **c** last Glacial Maximum

Occurrences of *B. hyrcana* were collected from the literature (Ahangaran 2016; Asadi et al. 2011, 2021; Esmailzadeh and Soleymanipor 2016; Esmaeilnezhad et al. 2020; Ghorbanalizadeh and Akhani 2022; Hosseinzadeh and Esmailzadeh 2017; Kakrodi et al. 2019; Khazaeli et al. 2018; Mohammadzadeh et al. 2019; Roodi et al. 2012; Salehi Shanjani et al. 2018; Soleymanipour and Esmailzadeh 2015) and the Hyrcanian Forest projects inventory of the Iranian Natural Resources and Watershed Management Organization (NRWO). Further, the “Georeferencer” option from QGIS software was used to estimate the populations without exact locations (QGIS Development Team 2020). Overall, 80 species occurrences were collected from the mentioned resources (Fig. 2a, Table S1).

Raster data with 19 bioclimatic variables (Table S2) for current conditions, future models, and the period of the Last Glacial Maximum (LGM, ca. 21 ka) were downloaded from the CHELSA database (Karger et al. 2017, 2018, 2021). The resolution of these rasters was 30 arc-sec. Three models were used for future conditions (climatic conditions in 2070): CCSM4 (Gent et al. 2011), MPI-ESM-P (Giorgetta et al. 2013), and MIROC-ESM (Watanabe et al. 2011). Estimations of the future range of species were conducted for three scenarios: RCP2.6 (optimistic, about + 1 °C before the end of XXI century), RCP 4.5 (moderate, about + 1.8 °C), and RCP 8.5 (pessimistic, about + 3.7 °C) (Collins et al. 2013). An additional set of rasters for past climate (Late Holocene, 4.2–0.3 ka) was downloaded from PaleoClim (Brown et al. 2018; Fordham et al. 2017). Pearson correlations between variables were calculated using the *layerStats* function from the package ‘raster’ in R (Hijmans and van Etten 2012; R Core Team 2021). To reduce collinearity between climatic factors, ten rasters with strong correlations (> 0.8) were dismissed.

MAXENT 3.3.3 k was used to calculate the theoretical range of species (Phillips et al. 2006). Each analysis was conducted as a bootstrap with 100 replications, 10,000 iterations, and 10^–5^ convergence thresholds. A random test partition was provided with a “random seed” and 20% localities as testing data. The ROC (Receiver Operating Characteristic) curve, TOC (Total Operating Characteristic) curve, and AUC (Area Under the Curve) values were used for model evaluation (Liu and Pontius Jr 2021; Mas et al. 2013; Wang et al. 2007). MAXENT output was visualized in QGIS 3.16.4 ‘Hanover’ (QGIS Development Team 2020).

CIRCUITSCAPE software was applied to outline the connectivity of topography and human population density in conjunction with *B.* stands formation of connectivity (McRae and Beier 2007). Two analyses have been prepared; altitude and human footprint rasters are considered resistance factors (Danielson and Gesch 2011; Venter et al. 2016, 2018). The results obtained were normalized, and a raster of the average resistance was created. A human footprint raster was used also to calculate the potential range of species (for current and future conditions) with a correction to the human population density, according to the formula: M*(1-H), where M and H were the suitability and H normalized value of human footprint raster, respectively. Obtained rasters were used in further analyses. Range fragmentation was estimated using the GuidosToolbox software with two methods: Normalized Hypsometric Curve and Entropy Map (Vogt and Riitters 2017). The morphological-spatial analysis (MSPA) implemented in the same software was used to measure the structure of the species' potential range in the study area (Soille and Vogt 2009). This type of analysis allows us to categorize the area into different classes such as range core, bridges, or edges.

## Results

### Model performance and contributions by variables

High AUC values were obtained from both the area under the ROC curve (0.989) and the area under the TOC curve (0.992), indicating that the models fit the data well (Fig. S1). The two most important bioclimatic variables in regional modeling are 1) Precipitation of Driest Month (bio14) with an average contribution of 77.9%, indicating that a suitable range of boxwood requires a humid climate which increases the probability of the presence; 2) Minimal Temperature of Coldest Month (bio6) (11.5%), which is a limiting factor in the high altitudes of the Alborz Mountains (Fig. S2, Table 1). Other bioclimatic variables (bio 3, 4, 5, 8, 9, 15, and 19) contributed less (below 10%).

**Table 1:** The contribution of variables (%) used to predict potential range of *Buxus hyrcana*

Bioclimatic variables	Climate scenarios							Average
	Current	RCP 2.6	RCP 4.5	RCP 8.5	LGM	LH	Current	
Isothetmality	bio3	1.4	1.1	1.1	1.2	1.3	0.8	**1.1**
Temperature Seasonality	bio4	3.4	3.6	3.4	3.1	3.6	4.4	**3.6**
Max Temperature of Warmest Month	bio5	0.1	0.1	0.2	0.2	0.1	0.0	**0.1**
Min Temperature of Coldest Month	bio6	11.5	11.3	11.4	11.2	10.0	10.2	**10.9**
Mean Temperature of Wettest Quarter	bio8	0.9	0.7	0.9	1.0	0.9	0.9	**0.9**
Mean Temperature of Driest Quarter	bio9	0.3	0.4	0.4	0.4	0.3	1.5	**0.5**
Precipitation of Driest Month	bio14	77.1	77.9	77.7	78.7	78.6	77.7	**77.9**
Precipitation Seasonality	bio15	1.5	1.5	1.8	1.3	1.4	1.6	**1.5**
Precipitation of Coldest Quarter	bio19	3.7	3.3	3.2	3.0	3.8	2.8	**3.3**

Average values are in bold as they are most significant

### Potential habitat under current climate change

The potentially suitable habitats of *B. hyrcana* were mainly distributed on the southern coast of the Caspian Sea and occupy western (Gilan), central (Mazandaran), and eastern (Golestan) parts of the Caspian coastal plain with an area of 38,699.24 km^2^ and 745.36 m a.s.l. average altitude (Fig. 2a, Table 2). The range edges are located in the Talysh Mountains in Azerbaijan and the westernmost part of Golestan. 12,441.64 km^2^ out of the total potential range is estimated as highly suitable (> 0.5) (Table 2).

**Table 2:** Potential suitable habitat distribution (km^2^) are tested scenarios and classification ranges under future climate scenarios

Climate Condition	Suitability habitat (km^2^)					Potential range			
	Weak (0.05–0.25)	Medium (0.25–0.5)	High (> 0.5)	Total	% of current area	Loss	Stable	Gain	% of Loss
LGM	28,367.57	10,305.04	6611.62	45,284.22	117%				
LH	14,865.74	14,182.33	6983.75	36,031.82	93%				
Current	15,588.61	10,668.99	12,441.64	38,699.24	100%				
RCP 2.6	20,310.56	9020.81	334.41	29,665.78	77%	10,738.62	27,960.62	1,705.16	28%
RCP 4.5	19,054.39	4118.45	0.00	23,172.84	60%	16,725.48	21,973.76	1,199.08	43%
RCP 8.5	11,903.79	0.00	0.00	11,903.79	31%	27,292.62	11,406.62	497.17	71%

### Potential habitat under past climate change

The potential range of *B. hyrcana* during the Last Glacial Maximum was 117% of the current area (Table 2), however, the spatial distribution of suitable areas was different (Fig. 2c). MAXENT estimated the existence of two highly desirable climatic refugia, in the western (Gilan) and central part (Mazandaran), by an area of 45,284.22 km^2^ during the LGM throughout the Hyrcanian forests; highly suitability area of 6,611.62 km^2^ was detected (Table 2). Contact zones do not occur due to barriers created by the Caspian Sea and Alburz Mountains. Interestingly, the central refugium areas by average altitude (1276.16 m a.s.l.) were located higher compared to current conditions (Fig. S3). In the later period, during the Late Holocene, the potential range of species started resembling the current range in case of the area (36,031.82 km^2^) and average altitude (758.88 m a.s.l.) (Figs. 2b, S3; Table 2).

### Potential habitat under future climate change (2070)

**Fig. 3:**
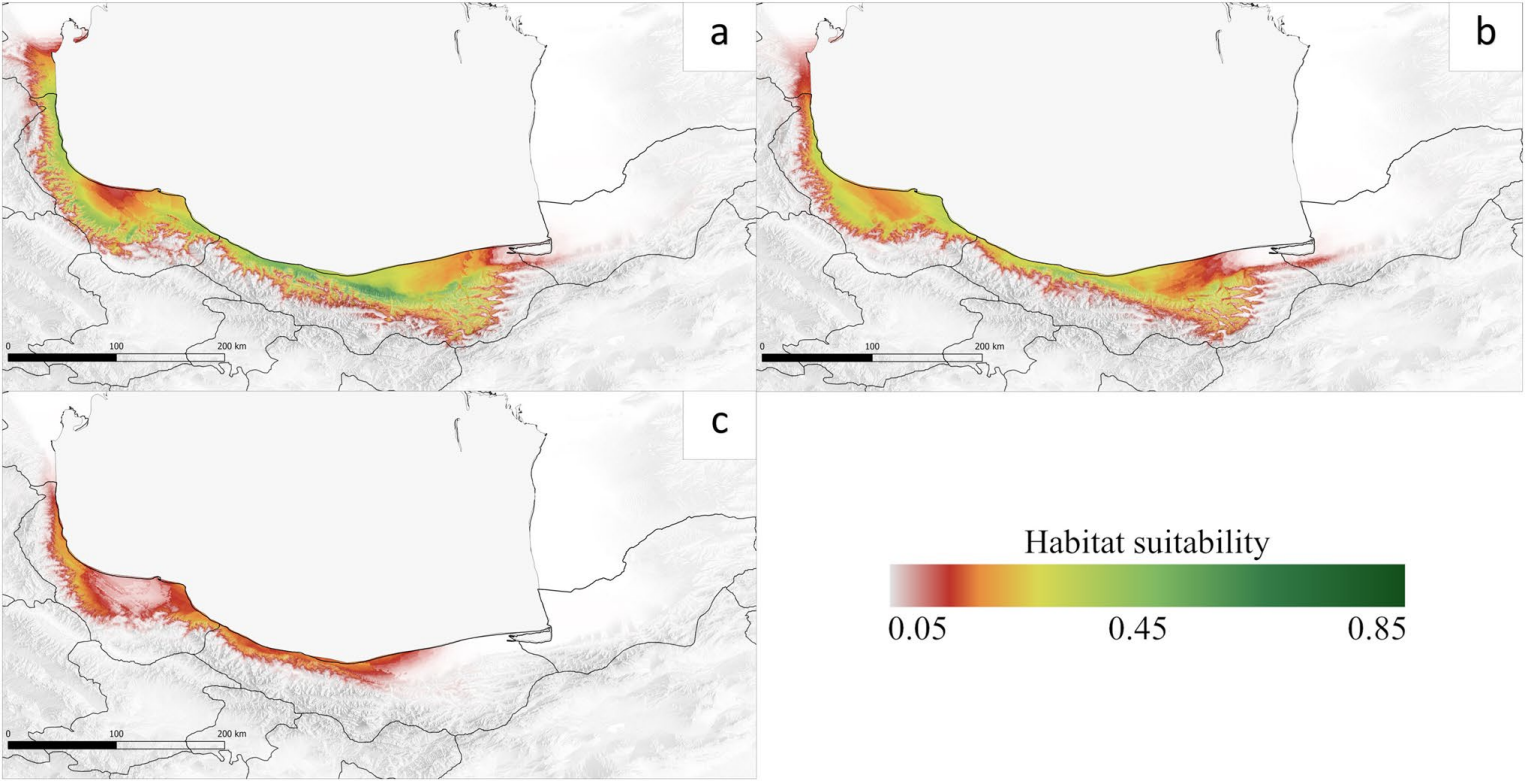
Average predicted distribution range of *Buxus hyrcana* in the future climatic scenarios for 2070. **a** RCP 2.6 scenario, **b** RCP 4.5 scenario, **c** RCP 8.5 scenario

In the optimistic future scenario (RCP 2.6), MAXENT estimated a significant reduction of the suitability habitat *B. hyrcana* by 77% of the current area in the whole of Hyrcanian Forests (Fig. 3a). There was a similar changing disposition in the distribution under the RCP2.6 and RCP4.5 scenarios with the difference being that range reductions are visible especially in the eastern part of Mazandaran and in Golestan and has a minimal expansion trend (Fig. 3a, b). It is explicit that the altitudinal range of the species will shift significantly in the future; under RCP4.5 and 8.5 the average altitude is projected as 472.53 m a.s.l. and 286.30 m a.s.l., respectively (Fig. S3). In the most pessimistic scenario RCP8.5, *B. hyrcana* would be faced with a trend of contraction and diminution expansion of the range (Fig. 3c). The area of high and medium suitability will disappear and only the less suitable habitat of 11,903.79 km2 will remain (Table 2). The potential range of *B. hyrcana* was classified into three levels: stable, loss and gain, shown in Table 2. Under the future climate change scenarios in the three periods, the stable habitats have occupied the western (Gilan) and the narrow longitudinal strip between the Alborz mountains and Caspian (Mazandaran) (Fig. S4). Furthermore, under RCP2.6 and 4.5 scenarios there is a possible gain of new areas of 1705.16, and 1199.08 km^2^, respectively. The Maximum loss of suitable habitat of *B. hyrcana* was predicted under RCP8.5 then 4.5 with 71% and 43%, respectively (Table 2).

### Potential range with correction on human population density

**Fig. 4:**
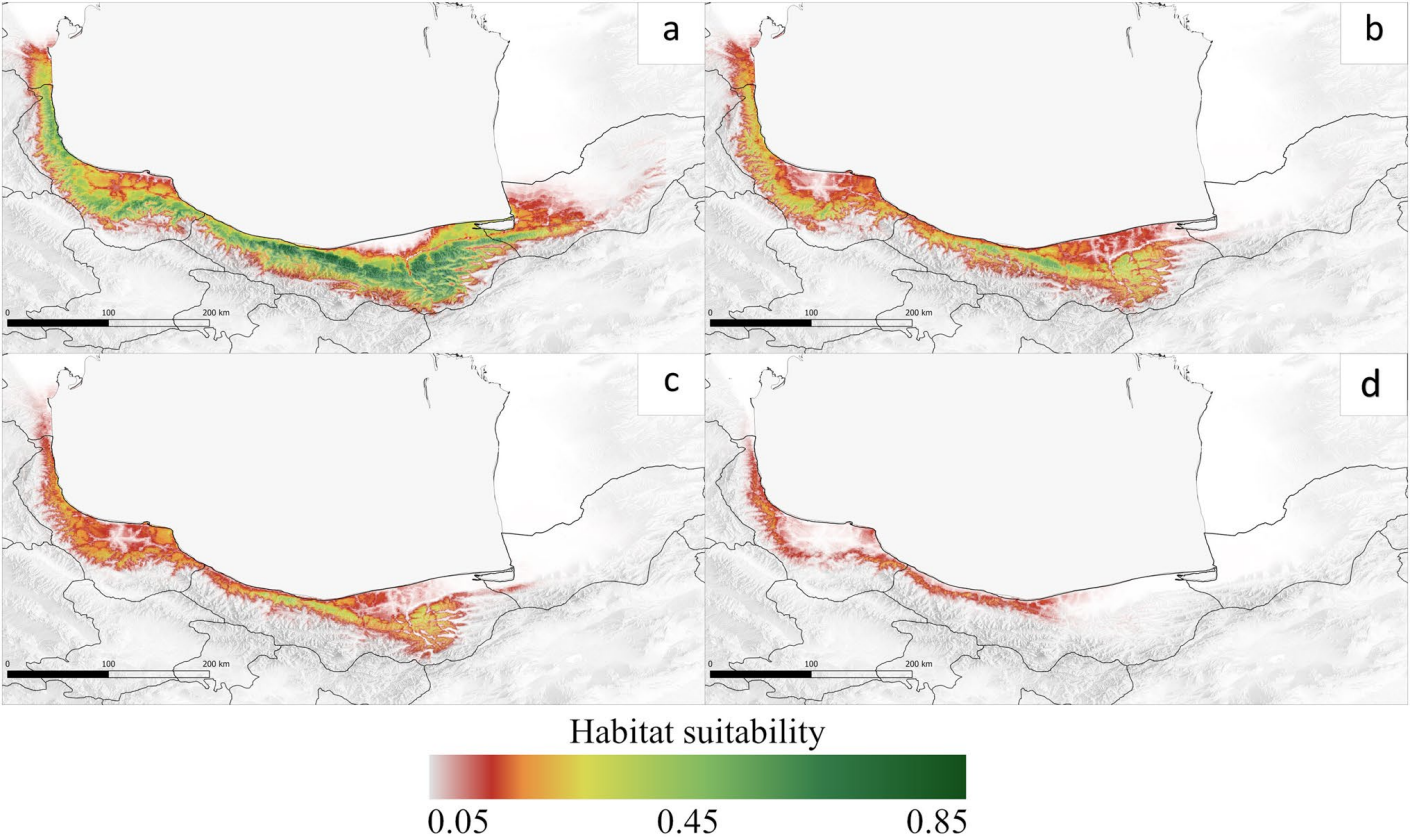
Current and future potential species distribution with correction on human population density. **a** Current; **b** RCP 2.6; **c** RCP 4.5; **d** RCP 8.5. The highest and lowest suitability in green and red colors, respectively

In Fig. 4 the potential distribution of *B. hyrcana* under human population density impact in different climate conditions was presented. In Fig. 4a the simulated human footprint intensifies parallel with the high-suitability area in the current condition. The simulated distribution pattern under future scenarios was even more human-influenced. Particularly for RCP8.5, the impact is very pronounced and the potential range of *B. hyrcana* becomes quite narrow throughout the Hyrcanian forests. Habitat changes attributed to human influence were one of the dominant contributors, as shown in Fig. 4d.

### Spatial connectivity model

CIRCUITSCAPE analysis has created the connectivity model using altitude and human footprint as resistance rasters to predict how those variables drive connectivity of particular parts of the range. The important movement routes and pathways connecting are identified in the central *B. hyrcana* habitats (Mazandaran) including more locations. In contrast, in the western (Gilan) and Eastern (Golestan) connectivity and potential corridors are disturbed because barriers exist (Fig. S5).

### MSPA model

**Fig. 5:**
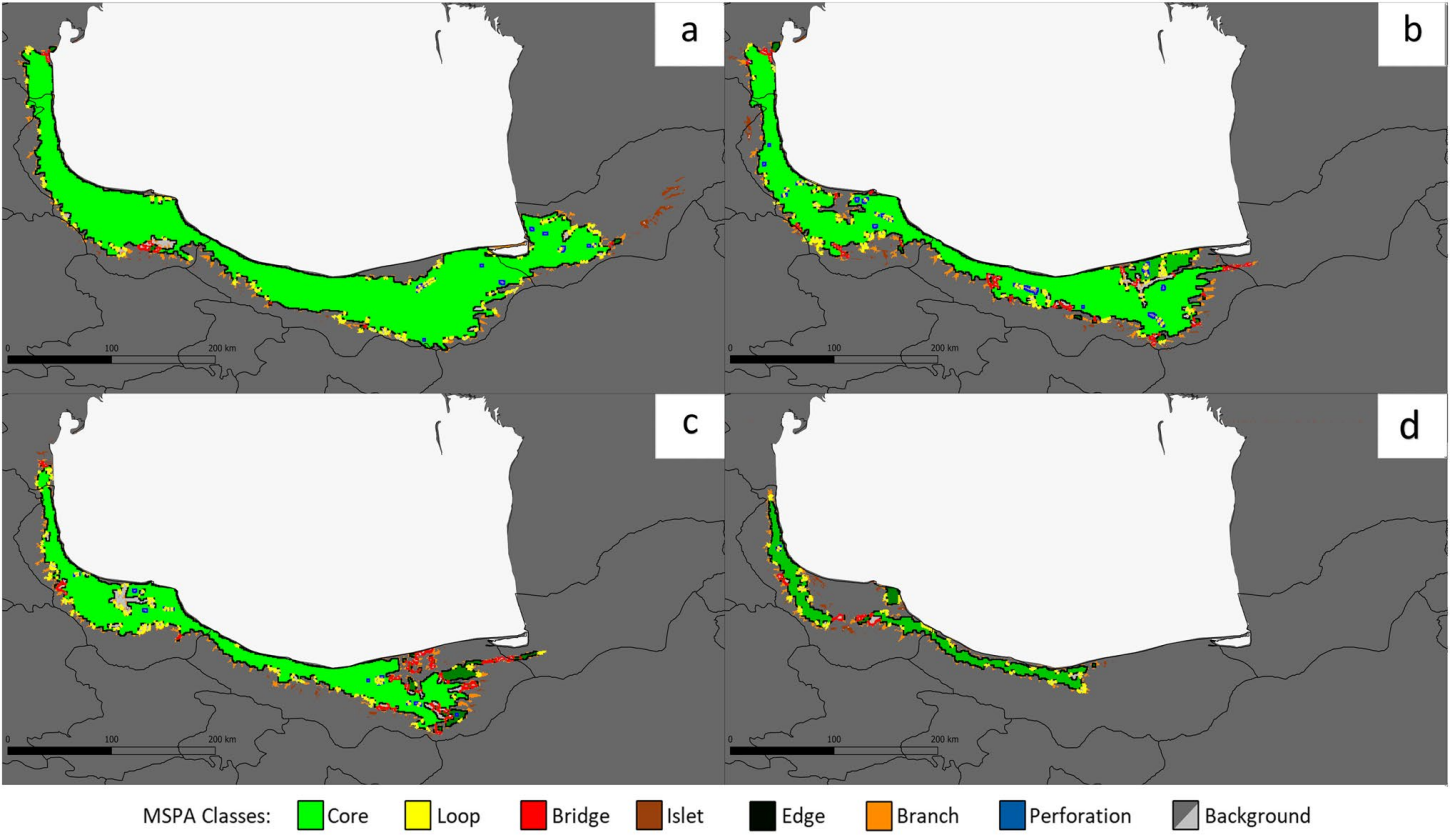
Spatial patterns of species range in the Hyrcanian zone under current conditions (**a**) and in future scenarios (**b** RCP 2.6; **c** RCP 4.5; **d** RCP 8.5)

Linear elements (potential connectivity) obtained by morphological spatial analysis (MSPA) according to the potential range of species with correction for human population density are shown in Fig. 5. In the present condition, core areas for potential species occurrence and adjacent patches of suitable habitat were detected throughout the Hyrcanian. These core areas are currently very well connected (Fig. 5a). In scenarios RCP2.6 and 4.5, there is a somewhat similar transformation; connectivity is reduced and fewer corridors and other linear features (loops, bridges, islands, and branches) are observed (Fig. 5b, c). In the RCP8.5 scenario, the most striking changes are projected, and the potential core area is limited to a narrow strip from Mazandaran to Gilan, especially in the northwest direction, which has been connected by several bridges, and the remaining areas are disconnected (Fig. 5d).

**Fig. 6:**
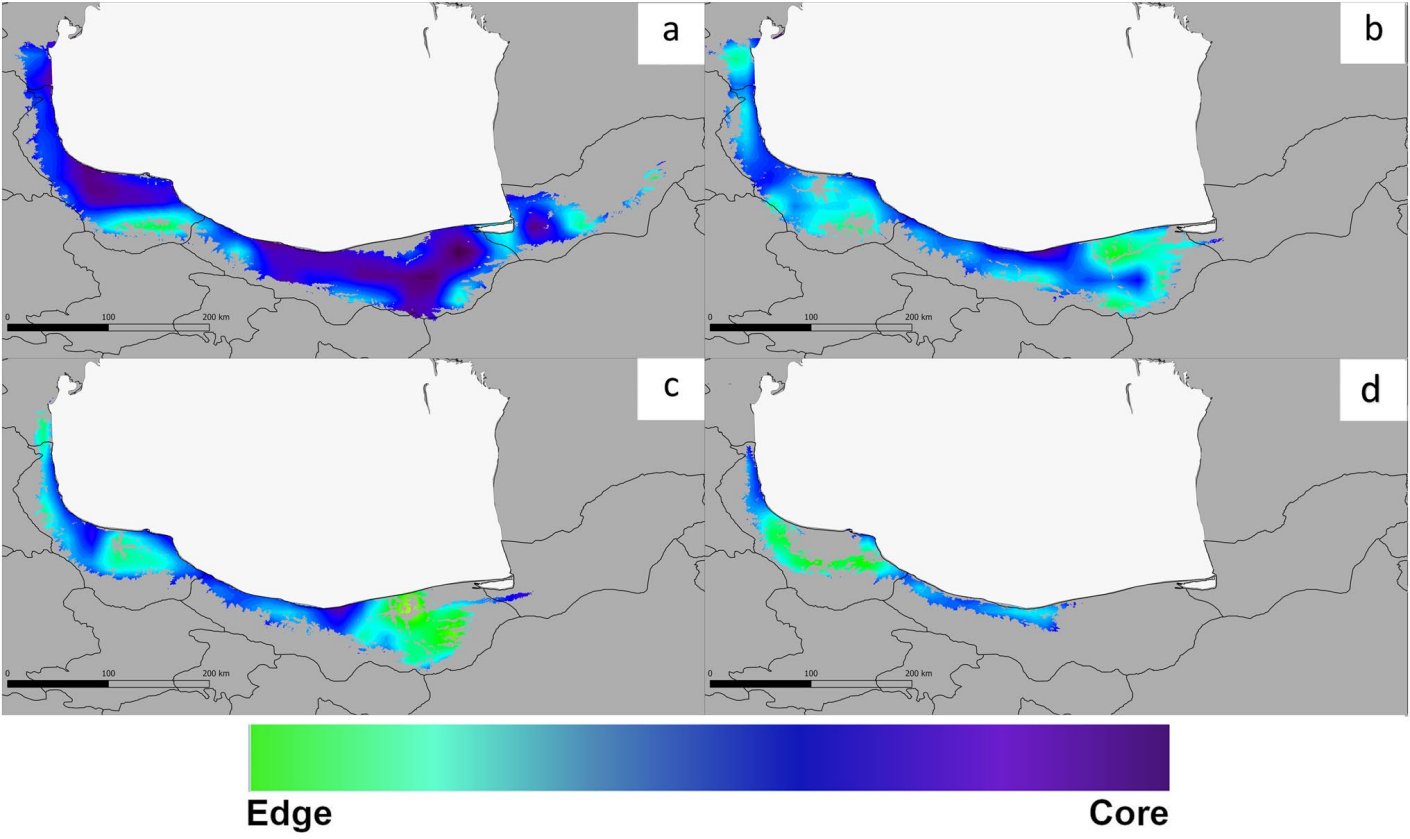
Pattern of edge/core ratio under the current (**a**), and future scenarios (**b** RCP 2.6; **c** RCP 4.5; **d** RCP 8.5. Range fragmentation in current conditions is 25.14%, whereas in the future scenarios 46.92%, 53.04% and 41.48%, respectively

Figure 6 represents patterns and processes of fragmentation. The results showed that the core patches with the best connectivity, resistance, and significantly lower values of fragmentation (25.14%) are mainly concentrated in the western, central, and slightly eastern part of the study area under the current climate condition, than in other climatic scenarios (Fig. 6a). Under optimistic and pessimistic future scenarios, there are substantial regional differences in habitats fragmentation among western, central, and eastern; a lack of core patches, high values of edge/core ratio, and expansion of the fragmentation (up to 53.04%) are predicted throughout the species distribution in Hyrcanian zone (Fig. 6b–d).

## Discussion

In the past, species have traveled long distances unimpeded in response to climate change (Sanderson et al. 2002; Schloss et al. 2012). However, adaptation and response to environmental change may become increasingly problematic in the future, as habitat fragmentation due to anthropogenic activities may hinder the ability of species to disperse long distances and shift their ranges (Keeley et al. 2018). Approximately one-third of the world's vascular plants are on the verge of extinction due to catastrophic human activities (Ren and Duan 2017), and one-third of tree species are threatened (Rivers et al. 2015). Therefore, conservation strategies for vulnerable species should include a comprehensive approach, increasing conservation areas, protecting climate refugia, rehabilitating habitats to adapt to climate change, and maintaining and enhancing landscape connectivity (Choe et al. 2018, 2020; Choudhury and Khan 2010). Additionally, maintaining connectivity by finding and protecting connections across the landscape (corridors, ecological networks, and roadways) is an important strategy and is essential to facilitate species movement to track relevant climates and species range shifts (Hilty et al. 2006; Keeley et al. 2018; Nuñez et al. 2013).

The results of the study showed a significant impact of human population density, in parallel with climate change, on the potential range of *B. hyrcana*. These factors have a strong impact on accelerating deforestation, fragmentation and forest degradation. The expansion of economic development and population growth in the Hyrcanian region has been accompanied by local activity in rural and urban areas. This has simultaneously increased environmental destruction and the extent of agriculture, logging, livestock and mining industries (Shirvani et al. 2020). Large-scale agricultural planting and road networks require sufficient and well accessible land to meet the demand, and the effects of their expansion are well documented for different forest ecosystems like Hyrcanian forests (Ali et al. 2005; Shirvani et al. 2017). The lowland areas of northern Iran have been subject to overexploitation for a long time and thus the habitats of many species have been severely damaged and annihilated (Akhani et al. 2010). Such destruction has taken place in case of *B. hyrcana* stands (Sagheb-Talebi et al. 2014). Noticeably, between 1966 and 2016, spatial forest patterns shifted from "forest loss" to "fragmentation," and the MSPA results provide evidence of this phenomenon (Shirvani et al. 2020).

Caspian boxwood is currently under threat due to pests, diseases, human pressure, and climate change. Our work was prepared to determine how climatic factors may shape the potential range of this species in the Hyrcanian region. MAXENT result suggests that the most significant variable is the precipitation in the driest month. Morphological studies showed that the boxwood, which is an evergreen species, relies on leaf plasticity to overcome water scarcity (Yosefzadeh et al. 2009). However, during the driest part of the year, such adaptation is insufficient and water scarcity is the limiting factor, much more important than temperature. The southern coast of the Caspian Sea, where *Buxus* occurs, is characterized by high precipitation, but there is a west–east gradient in rainfall in the Hyrcanian region. The highest precipitation occurs in the western part, whereas Golestan is relatively dry. This fact is the reason why the eastern part of the range may become unsuitable in the near future. Although the current potential range of the species is continuous, two main cores can be distinguished: the western and central cores, connected to each other at the Mazandaran-Gilan boundary. There is also a small compact area in Golestan, but this is disappearing in subsequent scenarios.

The modern range of the species was formed during the Holocene, and during the Meghalayan period (4.2–0.3 ka) it was almost the same as the current. During the Last Glacial Maximum, the species probably survived in the area of two refugia that were potential sources of recolonization; one existed in the Gilan, whereas another in mountainous areas of Mazandaran. A similar pattern was observed for *G. caspica* (Yousefzadeh et al. 2022). However, there is no genetic evidence of clear east–west division, like in the case of Caspian poplar (*P. caspica* ) (Alipour et al. 2021); instead, a pattern is observed which is related to the distance of particular populations from the Caspian Sea (Esmaeilnezhad et al. 2020). It is possible that the species survived in only one refugium, or that two gene pools mixed and lost their distinctness; it is also possible that the model underestimated the suitability in the central part of the Hyrcanian and the range of the species during LGM was continuous.

In the near future, *B. hyrcana* stands will be endangered by the negative impact of environmental changes. Reduction in suitability is observed even in the most optimistic climate change scenario, whereas in the most pessimistic the whole range of the species is endangered. In scenario RCP 8.5, suitable areas constitute only 31% of the current potential range and form two narrow belts: one in the west Gilan and the second from the Gilan-Mazandaran border to the central coast of Mazandaran. Between these two potential refugia is an area of lowlands where conditions are less favorable for the species. The situation becomes even more disturbing if we consider human pressure. The southern coast of the Caspian Sea is densely populated and a big part of this region was transformed into crop fields (Sagheb-Talebi et al. 2014). The results of the analyses performed in GuidosToolbox software suggest that *Buxus* is at serious risk of range fragmentation in the future. Areas with suitable climatic conditions will not only shrink but also the connectivity between them will deteriorate. The analysis prepared in CIRCUITSCAPE with human footprint as a resistance raster further confirms the risk of fragmentation. The reason is not only human pressure, but also the spatial pattern of occurrence of the species, which prefers lowlands (Sagheb-Talebi et al. 2014). Thus, opportunities to change the altitudinal range are limited. Modeling results suggest even a loss of sites at higher elevations, probably due to a decrease in precipitation. Range fragmentation can be a major threat because, for most forest plant species, such a phenomenon severely impedes colonization and retreat to areas with a stable climate (Honnay et al. 2002).

Boxwood in Iran is currently endangered; therefore, there is a need to plan effective conservation strategies for this species. In 2016, a decision was made to limit the exploitation of the Hyrcanian forests in order to protect them (Panahi et al. 2021). Unfortunately, in many places this resulted in a shift in the exploitation of forest resources from formal to smuggling. The disappearance of suitable habitats for woody species is also associated with the expansion of cities and farmland on the Caspian coast. Recently, the spread of disease, boxwood blight (Kakrodi et al. 2019; Salehi Shanjani et al. 2018) and a pest, box-tree moth, *Cydalima perspectalis* (Walker) (Nacambo et al. 2014), have become an even greater threat to the boxwood. Human pressure and the prevalence of biological threats mean that in situ conservation would be difficult. One of the main actions that is taken to protect the species is to cut down and burn infected trees, which gives other individuals the opportunity to survive. Based on the results of genetic studies, Salehi Shanjani et al. (2018) suggest that focusing conservation efforts on surviving individuals in populations infested with boxwood blight may be a good strategy. There are also stands that are still free from the presence of the disease (Panahi et al. 2021). The modeling results indicate that there are two areas where the climate will be stable enough to host potential reserves: one in Gilan and the other along the west coast of Mazandaran. Unfortunately, populations from the eastern part of the range at higher elevations are at risk. Because they exhibit genetic distinctiveness from lowland populations, their loss would be a major impoverishment to the species' gene pool (Esmaeilnezhad et al. 2020). Although inland populations appear to be more vulnerable due to climate change, sites located near the Caspian Sea may be more susceptible to the disease, which is associated with higher moisture. However, protecting selected populations located in an area that will retain their suitability in the future seems to be a good strategy. An example of such a population might be the Siahkalrood site located near the coast, for which high genetic diversity has been demonstrated (Esmaeilnezhad et al. 2020). In addition, such protected populations may provide genetic enrichment to other sites, as pollen may be carried by winds along the Caspian coast (Esmaeilnezhad et al. 2020; Molavi-Arabshahi et al. 2016).

Another strategy for an endangered species is ex situ conservation. In the case of *B. hyrcana,* such efforts were undertaken by the National Botanical Garden of Iran (NBGI), where the species began to be cultivated as early as 1967 (Panahi et al. 2021). Currently, NGBI is the only reliable source of healthy seedlings of the *B. hyrcana* and can provide approximately 5,000 individuals per year; these seedlings can be used to restore damaged boxwood populations in Hyrcanian forests (Panahi et al. 2021). Unfortunately, so far there has been no documentation of the gene pool that is available at NBGI, which may pose some difficulty in planning reintroductions to degraded sites. Such attempts must also take into account the continued threat of boxwood blight and pests, so selected sites should first be properly managed for the presence of these biological threats. Efforts should be made to collect material from these populations, which may soon disappear due to climate change—these are primarily stands from Golestan and eastern Mazandaran.

## Conclusion

The results of our study indicate that *B. hyrcana* is one of the species whose range may be subject to reduction and fragmentation associated with climate change and human pressure. The analyses we performed in this work provide a better understanding of the transformation of the potential range of this endangered species. They can also provide guidance for in situ conservation planning in stable regions such as central Gilan and point to those areas where populations are likely to become extinct in the near future—such sites could become a source of material for ex situ conservation, in order to avoid loss of their genetic variability. Unfortunately, protecting Capsian boxwood, which is currently threatened by pests, diseases, climate change, and human pressure, will be a major challenge—it remains to be hoped that the strategies developed will be effective. The situation is all the more difficult because populations located in the mountains are particularly threatened by climate change, while those located by the sea are more likely to be attacked by the pest. The status of existing populations should be monitored, both in terms of climate impacts and pest infestation. Conducting additional analyses (for example, based on the recently published IPCC6 report) can further facilitate the planning of effective conservation methods for this endangered species.

## Supplementary Information

Below is the link to the electronic supplementary material.

Supplementary file1 (PDF 1089 KB)

## Data Availability

The data that support the findings of this study are available on request from the corresponding author.
