# Supplementary material for: The influence of climate and population density on Buxus hyrcana potential distribution and habitat connectivity
Source: J Plant Res. 2023 Apr 28;136(4):501–14. doi: 10.1007/s10265-023-01457-5 (PMC10250272; doi:10.1007/s10265-023-01457-5)
Supplement: Supplementary file 1 — Supplementary file1 (PDF 1089 KB) [file 10265_2023_1457_MOESM1_ESM.pdf]

Electronic supplementary materials

Title: The influence of climate and population density on *Buxus hyrcana* potential distribution and habitat connectivity

Authors:

Shirin Alipour*1, Łukasz Walas*1

Journal:

Journal of Plant Research

Corresponding author:

Shirin Alipour*1, Łukasz Walas*1

1 Institute of Dendrology, Polish Academy of Sciences, Parkowa 5, 62-035 Kórnik, Poland

Tel: +48-78-0134893 (S. Alipour), +48-50-6630851 (Ł. Walas)

E-mail: salipour@man.poznan.pl; lukaswalas@man.poznan.pl;

Content:

Tables S1-S2

Figs. S1–S5

Table S1. Locations of stands used in MAXENT analysis.

N	x	y	Source
1	53.02222	36.29861	Asadi et al. 2011
2	53.07083	36.34722	Asadi et al. 2011
3	50.68793	36.77643	Asadi et al. 2021
4	51.12239	36.69984	Asadi et al. 2021
5	51.1725	36.6885	Asadi et al. 2021
6	52.01683	36.34197	Asadi et al. 2021
7	52.04047	36.28335	Asadi et al. 2021
8	52.10287	36.34008	Asadi et al. 2021
9	52.11138	36.23607	Asadi et al. 2021
10	52.82524	36.41194	Asadi et al. 2021
11	52.91601	36.40059	Asadi et al. 2021
12	52.9179	36.48096	Asadi et al. 2021
13	52.98786	36.52634	Asadi et al. 2021
14	53.00015	36.45921	Asadi et al. 2021
15	53.04554	36.40437	Asadi et al. 2021
16	53.16089	36.2356	Asadi et al. 2021
17	53.20627	36.18265	Asadi et al. 2021
18	53.21857	36.70079	Asadi et al. 2021
19	53.23369	36.26586	Asadi et al. 2021
20	53.28097	36.21669	Asadi et al. 2021
21	54.20945	36.75752	Asadi et al. 2021
22	53.1375	36.08361	Esmailzadeh & Soleymanipor 2016
23	53.45611	36.18472	Esmailzadeh & Soleymanipor 2016
24	53.81111	36.70333	Esmailzadeh 2020
25	53.87778	36.70833	Esmailzadeh 2020
26	48.83936	38.74496	Ghorbanalizadeh & Akhani 2022
27	48.85449	38.47266	Ghorbanalizadeh & Akhani 2022
28	49.03981	37.64062	Ghorbanalizadeh & Akhani 2022
29	49.5882	37.27754	Ghorbanalizadeh & Akhani 2022
30	49.62602	37.03171	Ghorbanalizadeh & Akhani 2022
31	49.98531	37.20758	Ghorbanalizadeh & Akhani 2022
32	49.99854	37.17732	Ghorbanalizadeh & Akhani 2022
33	50.20845	37.08466	Ghorbanalizadeh & Akhani 2022
34	51.34872	36.57504	Ghorbanalizadeh & Akhani 2022
35	51.40923	36.63555	Ghorbanalizadeh & Akhani 2022
36	51.51324	36.59962	Ghorbanalizadeh & Akhani 2022
37	51.57942	36.60151	Ghorbanalizadeh & Akhani 2022
38	51.83471	36.52398	Ghorbanalizadeh & Akhani 2022
39	52.3623	36.29139	Ghorbanalizadeh & Akhani 2022

40	52.88421	36.29328	Ghorbanalizadeh & Akhani 2022
41	52.91258	36.19684	Ghorbanalizadeh & Akhani 2022
42	53.01091	36.08527	Ghorbanalizadeh & Akhani 2022
43	53.51013	36.63271	Ghorbanalizadeh & Akhani 2022
44	53.52904	36.70646	Ghorbanalizadeh & Akhani 2022
45	54.47076	36.7197	Ghorbanalizadeh & Akhani 2022
46	52.27278	36.34167	Hosseinzadeh & Esmailzadeh 2017
47	51.00083	36.58667	Kakrodi et al. 2019
48	51.10444	36.6375	Kakrodi et al. 2019
49	48.79045	38.4236	Khazaeli et al. 2018
50	49.60374	36.99635	Khazaeli et al. 2018
51	49.84596	37.06868	Khazaeli et al. 2018
52	50.81989	36.66834	Khazaeli et al. 2018
53	50.85521	36.7003	Khazaeli et al. 2018
54	51.10164	36.66077	Khazaeli et al. 2018
55	51.46161	36.57246	Khazaeli et al. 2018
56	51.62561	36.55228	Khazaeli et al. 2018
57	51.70046	36.54639	Khazaeli et al. 2018
58	51.777	36.54891	Khazaeli et al. 2018
59	52.3447	36.32435	Khazaeli et al. 2018
60	52.83251	36.33445	Khazaeli et al. 2018
61	52.83587	36.21334	Khazaeli et al. 2018
62	52.85438	36.17801	Khazaeli et al. 2018
63	53.17902	36.20661	Khazaeli et al. 2018
64	53.28667	36.60863	Khazaeli et al. 2018
65	53.84513	36.70451	Khazaeli et al. 2018
66	53.88333	36.75	Mohammadzadeh et al. 2019
67	54.33333	36.76667	Mohammadzadeh et al. 2019
68	48.95	37.72639	Personal communication, Shirin Alipour
69	49.03447	37.67187	Personal communication, Shirin Alipour
70	49.15417	37.54778	Personal communication, Shirin Alipour
71	49.57639	37.01139	Personal communication, Shirin Alipour
72	49.7575	37.37472	Personal communication, Shirin Alipour
73	50.89611	36.67667	Personal communication, Shirin Alipour
74	51.81037	36.57711	Personal communication, Shirin Alipour
75	53.32392	36.04758	Personal communication, Shirin Alipour
76	51.78333	36.55833	Roodi et al. 2012
77	49.55	37.01667	Salehi Shanjani et al. 2018
78	51.73333	36.56667	Salehi Shanjani et al. 2018
79	53.1375	36.01806	Soleymanipour & Esmailzadeh 2015
80	51.30028	36.68944	Ahangaran 2016

Ahangaran, Y. (2016). 'The first report of the Box Tree Moth from Iran, Cydalima perspectalis (Walker, 1859) Lep; Crambidae (short report)', Applied Entomology and Phytopathology, 84(No. 1), pp.

209-211.

Asadi, H., Esmailzadeh, O., De Cáceres, M., & Hosseini, S. M. (2021). The assignment of relevés to pre-existing vegetation units: a comparison of approaches using species fidelity. Annals of Forest Science, 78(1), 1-23.

Asadi, H., Hosseini, S. M., Esmailzadeh, O., & Ahmadi, A. (2011). Flora, Life form and chorological study of Box tree (Buxus hyrcana Pojark.) sites in Khybus protected forest, Mazandaran. Iranian Journal of Plant Biology, 3(8), 27-40.

Esmailzadeh, O. (2020). Classification of box tree plant communities (Buxus hyrcana Pojark.) In the Cheshmeh bolbol forest (Bandar Gaz Golestan). Journal of Forest Research and Development, 6(3), 491-503.

Esmailzadeh, O., & Soleymanipor, S. S. (2016). Improving the ordination of ecological species group using diagnostic species concept. Forest and Wood Products, 69(3), 495-509.

Ghorbanalizadeh, A., & Akhani, H. (2022). Plant diversity of Hyrcanian relict forests: An annotated checklist, chorology and threat categories of endemic and near endemic vascular plant species. Plant diversity, 44(1), 39-69.

Hosseinzadeh, S., & Esmailzadeh, O. (2017). Floristic study of Buxus hyrcana stands in the western forests of Haraz district, Amol. Iranian Journal of Applied Ecology, 6(1), 1-13.

Kakrodi, R. K., Kiadaliri, H., Mattaji, A., Nimvari, M. E., & Sheykholeslami, A. (2019). Box tree (Buxus hyrcana Pojark) habitat investigation for lichen and herbaceous community after disturbance caused by box blight disease (case study: Lesakoti Forest, Tonekabon). Iranian Journal of Forest and Poplar Research, 27(1).

Khazaeli, P., Rezaee, S., Mirabolfathy, M., Zamanizadeh, H., & Kiadaliri, H. (2018). Genetic and phenotypic variation of Calonectria pseudonaviculata isolates causing boxwood blight disease in the Hyrcanian forest of Iran. Agricultural Research & Technology: Open Access Journal, 19(1), 556081.

Mohammadzadeh, A., Payamnoor, V., & Kavosi, M. (2019). Evaluation type of explants and season of sampling under different disinfection treatments for the tissu culture of Buxus hyrcana Pojark. Journal of Forest Research and Development, 5(4), 527-540.

Roodi, Z., Jalilvand, H., & Esmailzadeh, O. (2012). Edaphic effects on distribution of plant ecological groups (Case study: Sisangan Buxus (Buxus hyrcana Pojark.) forest reserve). Iranian Journal of Plant Biology, 4(13), 39-56.

Salehi Shanjani, P., Javadi, H., Rasoulzadeh, L., & Amirkhani, M. (2018). Evaluation of genetic differentiation among healthy and infected Buxus hyrcana with boxwood blight using RAPD and ISSR markers. New Zealand Journal of Forestry Science, 48(1), 1-10.

Soleymanipour, S. S., & Esmailzadeh, O. (2015). Flora, life form and chorology of Box trees (Buxus hyrcana) habitats in forests of the Farim area of Sari. Taxonomy and Biosystematics, 7(23), 39-56.

Table S2. Tested bioclimatic variables.

Abbreviation	Description
**bio1**	Annual mean temperature
**bio2**	Mean Diurnal Range of Temperature
**bio3**	Isothetmality
**bio4**	Temperature Seasonality
**bio5**	Max Temperature of Warmest Month
**bio6**	Min Temperature of Coldest Month
**bio7**	Temperature Annual Range
**bio8**	Mean Temperature of Wettest Quarter
**bio9**	Mean Temperature of Driest Quarter
**bio10**	Mean Temperature of Warmest Quarter
**bio11**	Mean Temperature of Coldest Quarter
**bio12**	Annual Precipitation
**bio13**	Precipitation of Wettest Month
**bio14**	Precipitation of Driest Month
**bio15**	Precipitation Seasonality
**bio16**	Precipitation of Wettest Quarter
**bio17**	Precipitation of Driest Quarter
**bio18**	Precipitation of Warmest Quarter
**bio19**	Precipitation of Coldest Quarter

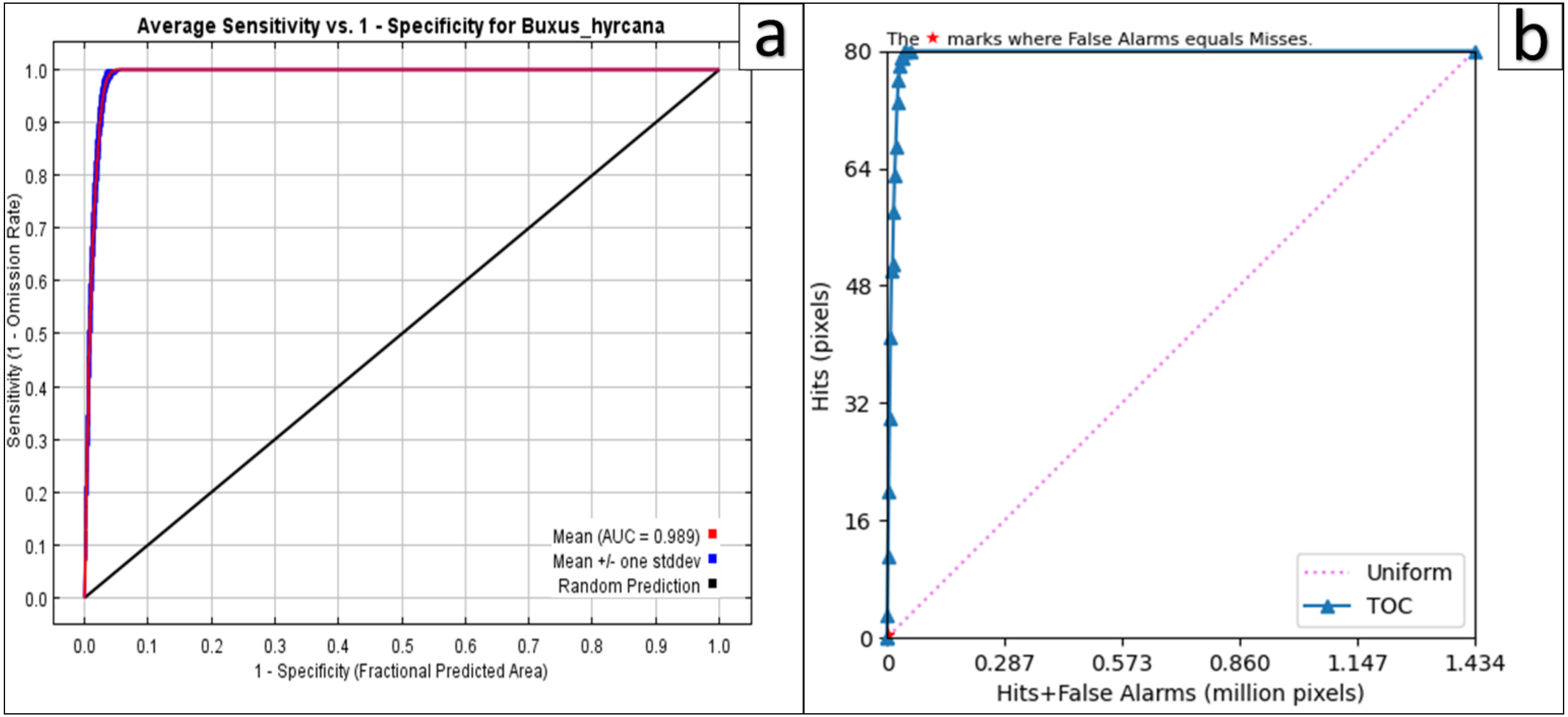

Fig S1. Accuracy of current climate model according to: a) ROC curve b) TOC curve.

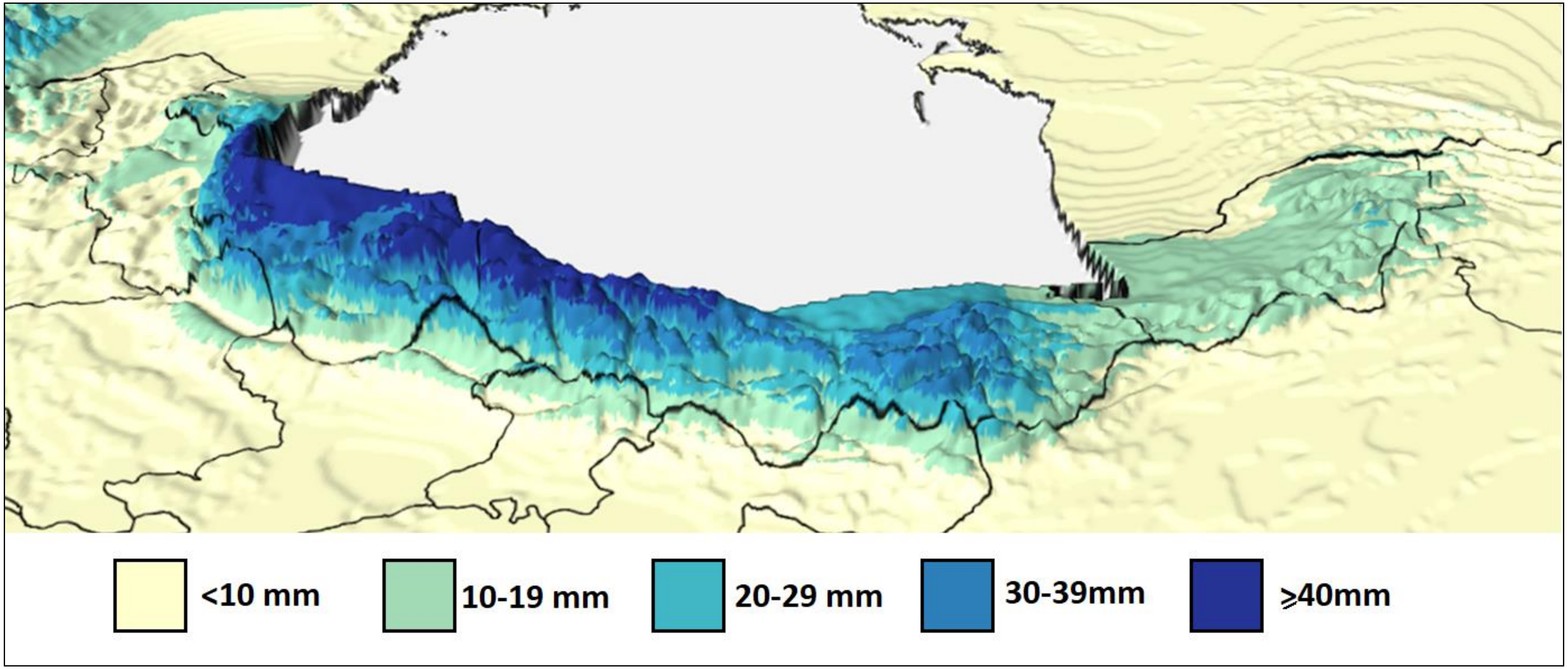

Fig S2 . Precipitation of driest month. Colors and height indicate values of rainfall.

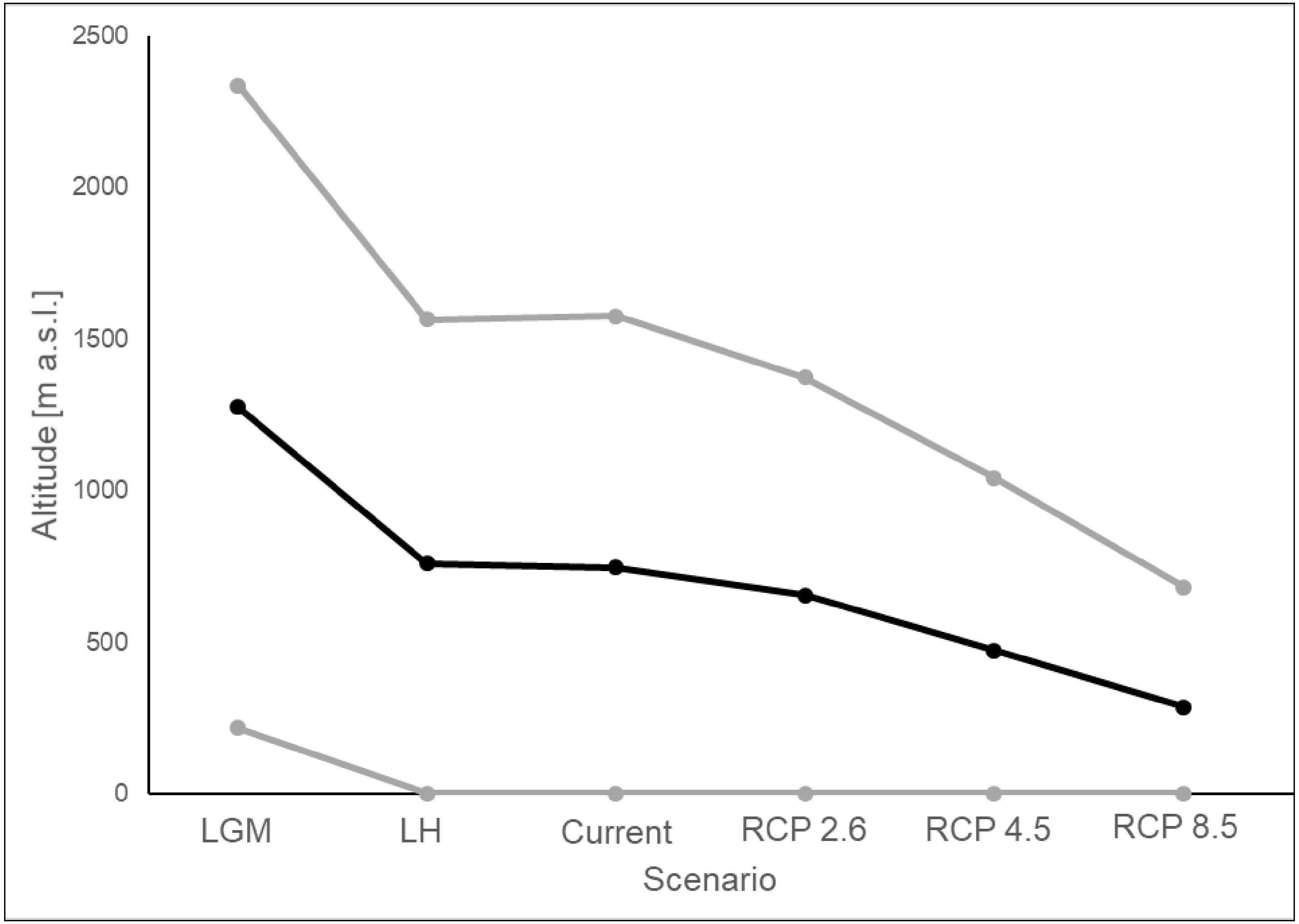

Fig S3. Changes in average altitude (black line) with standard deviation (gray lines).

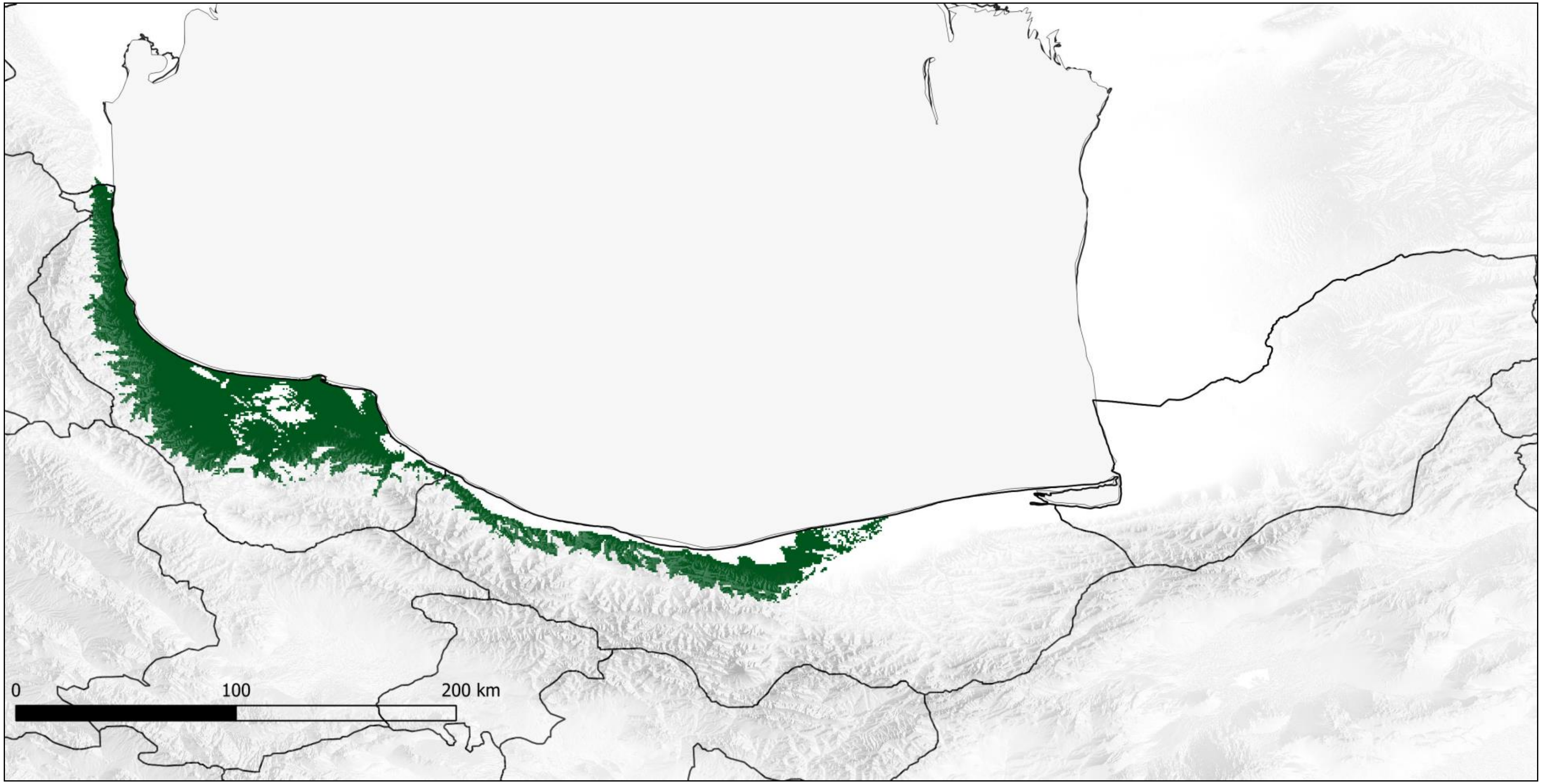

Fig S4. Stable areas with suitability higher than 0.05 in all tested scenarios (green color).

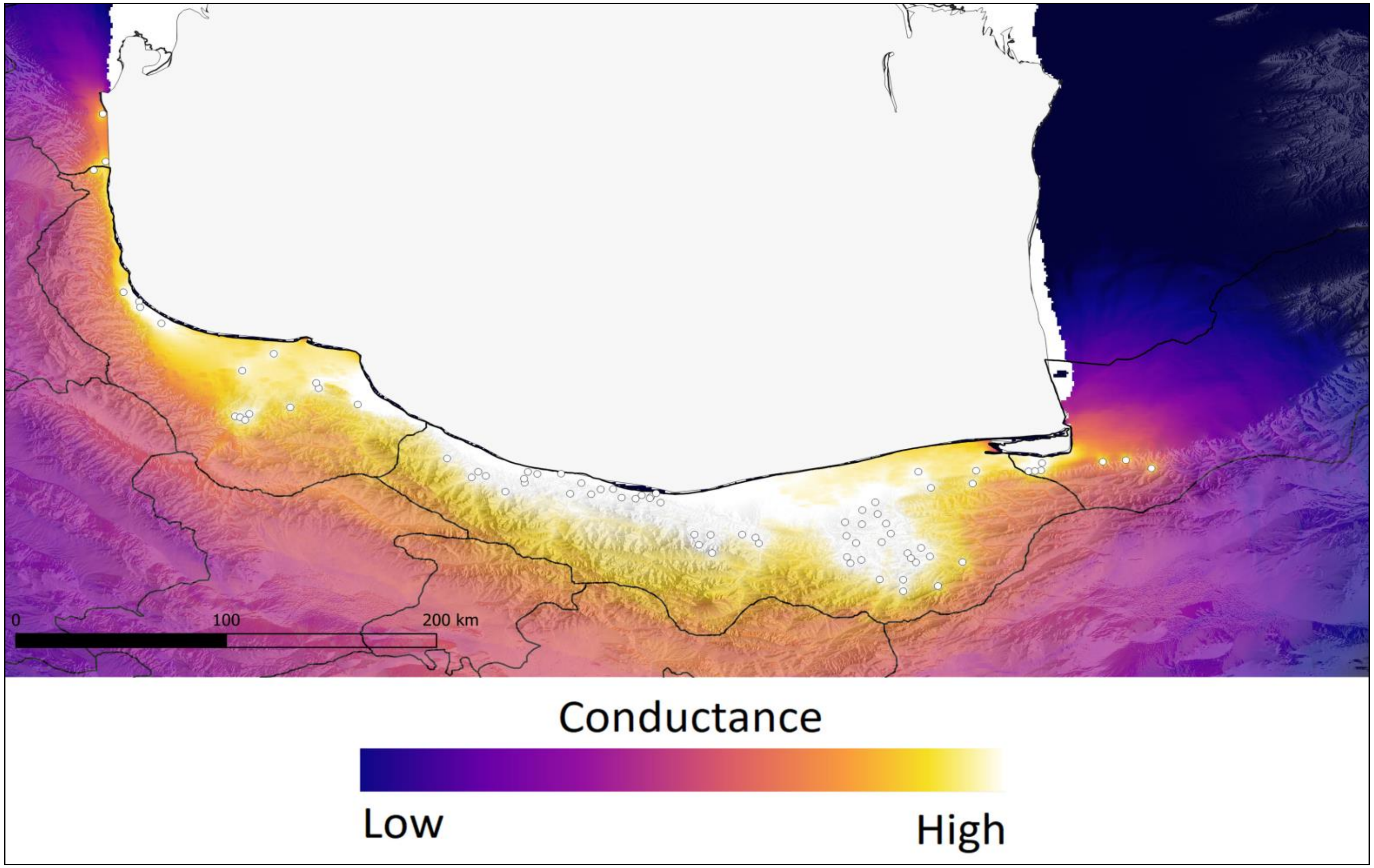

Fig S5. Conductance analysis with two rasters of resistance: altitude and human population density; the highest and lowest conductivity in white and dark, respectively. Dots indicated *B. hyrcana* locations.
